# Chronic Venous Disease and In-Hospital Venous Thromboembolism in Critically Ill Patients: A Retrospective Cohort Study of the MIMIC-IV Database

**DOI:** 10.3390/jcm15176695

**Published:** 2026-08-28

**Authors:** Alperen Kutay Yıldırım, Mesher Ensarioğlu

**Affiliations:** 1 Department of Cardiovascular Surgery, Gülhane Training and Research Hospital, University of Health Sciences, 06010 Ankara, Turkey; drayildirim@yahoo.com; 2 Department of Anesthesiology and Reanimation, Gülhane Training and Research Hospital, University of Health Sciences, 06010 Ankara, Turkey

**Keywords:** chronic venous disease, chronic venous insufficiency, varicose veins, CEAP classification, venous thromboembolism, intensive care, thromboprophylaxis, MIMIC-IV, observational study

## Abstract

**Background:** Chronic venous disease (CVD), and varicose veins in particular, has been reported as a risk factor for venous thromboembolism (VTE) in ambulatory and surgical populations, but its independent contribution to VTE risk in critically ill patients has not been studied. **Methods:** A retrospective cohort study was performed using the Medical Information Mart for Intensive Care-IV (MIMIC-IV) database, version 3.1. Adult patients with an intensive care unit (ICU) admission and length of stay ≥48 h were eligible. CVD, spanning Clinical-Etiological-Anatomical-Pathophysiological (CEAP) classes C2–C6, was ascertained from ICD-9 and ICD-10 codes using a cross-admission look-back. The primary outcome was in-hospital VTE. Multivariable logistic regression was performed with adjustment for age, sex, severity scores, comorbidities, and ICU treatments; additional analysis was performed with propensity score matching (1:3 nearest-neighbor) and multiple imputation for missing body mass index (BMI). **Results:** Of 28,741 eligible patients, 544 (1.9%) had documented CVD. Crude VTE incidence was 7.0% in CVD patients vs. 6.2% in those without CVD (unadjusted OR 1.14, 95% CI 0.82–1.59; adjusted OR 1.10, 95% CI 0.77–1.52, *p* = 0.571). After multiple imputation for BMI, the adjusted association attenuated to 1.03 (0.74–1.45, *p* = 0.849). Propensity score matching produced consistent results (OR 1.24, 0.84–1.84). In an exploratory severity analysis, crude VTE rates were 6.2% without CVD, 6.4% with uncomplicated CVD, and 16.7% with complicated CVD; the complicated stratum comprised only 30 patients with 5 events (aOR 2.90, 95% CI 1.09–7.08, *p* = 0.032), and the test for trend was not significant (*p* = 0.31). CVD patients received less pharmacological prophylaxis than non-CVD patients (61.2% vs. 66.0%, *p* = 0.023). **Conclusions:** Chronic venous disease was not independently associated with in-hospital VTE in this critically ill cohort, and the modest crude association attenuated further after accounting for BMI. An exploratory analysis suggested a higher risk in complicated CVD, but the stratum was too small and the trend test too weak to support a severity gradient; this requires confirmation in cohorts with prospective CEAP classification. CVD patients received less thromboprophylaxis than patients without CVD (61.2% vs. 66.0%, *p* = 0.023), although this difference did not persist after adjustment (aOR 0.88, 95% CI 0.74–1.06, *p* = 0.172).

## 1. Introduction

Venous thromboembolism (VTE), comprising deep vein thrombosis (DVT) and pulmonary embolism (PE), is a major cause of preventable morbidity and mortality among hospitalized patients [1,2]. Critically ill patients have a high burden of VTE; despite thromboprophylaxis, the reported incidence of VTE in intensive care unit (ICU) cohorts ranges from approximately 5% to 15% [3,4]. International guidelines recommend routine pharmacological or mechanical prophylaxis for ICU patients, yet substantial variation in implementation persists across institutions and patient subgroups [5,6]. Although well-established risk factors such as immobility, mechanical ventilation, vasopressor support, advanced age, malignancy, and recent surgery are routinely integrated into clinical decision-making and into scoring systems such as the Caprini score, developed in surgical inpatients, and the Padua score, developed in hospitalized medical patients, neither of which was derived specifically for the intensive care setting, the contribution of chronic venous disease has received considerably less attention in the ICU setting [7,8].

Chronic venous disease (CVD) denotes the full spectrum of long-standing morphological and functional abnormalities of the lower-limb venous system, graded in the Clinical-Etiological-Anatomical-Pathophysiological (CEAP) classification from telangiectasias and reticular veins (C1) and varicose veins (C2) through edema (C3), skin changes (C4), and healed or active venous ulceration (C5–C6) [9,10]. By transatlantic consensus, the narrower term chronic venous insufficiency (CVI) is reserved for the advanced end of that spectrum (C3–C6), in which a functional venous abnormality produces edema, skin changes, or ulceration [11]. The distinction is not merely semantic. In the population-based Edinburgh Vein Study, trunk varices were present in 40% of men and 32% of women, whereas CVI affected 9% of men and 7% of women [12]. CVD at every stage becomes more frequent with advancing age and with obesity [10,13,14]. Its pathophysiology involves venous hypertension, valvular incompetence, inflammation of the venous wall, and progressive endothelial dysfunction [15,16]. These processes are plausible contributors to thrombus formation because the venous stasis, impaired endothelial regulation, and pro-inflammatory milieu that characterize CVD overlap with the components of Virchow’s triad [17].

A study by Chang and colleagues, using Taiwanese national insurance data, demonstrated a strong association between varicose veins and incident VTE, with adjusted hazard ratios of 5.30 for DVT and 1.73 for PE among 213,000 propensity-matched pairs followed for over 7 years [18]. Subsequent investigations have largely supported this association across specific populations. A 2023 meta-analysis of patients undergoing lower-limb arthroplasty found that pre-existing varicose veins conferred a 2.4-fold increase in the odds of postoperative VTE [19]. Colorectal cancer surgery carries a substantial postoperative VTE risk for which dedicated prediction models have been developed [20]. Among hospitalized patients with acute exacerbation of chronic obstructive pulmonary disease (AECOPD), varicose veins were identified as a sex-specific risk factor in male patients (odds ratio 6.17, 95% CI 3.24–11.76) [21]. The consistency of these findings across diverse hospitalized populations suggests that chronic venous disease may contribute to in-hospital VTE risk [22]. It is notable that each of these studies used varicose veins, defined as CEAP C2 disease rather than chronic venous insufficiency, as the exposure, so the available evidence concerns chronic venous disease broadly rather than advanced venous disease specifically.

To our knowledge, no study has specifically evaluated the contribution of CVD to in-hospital VTE among unselected critically ill patients. Whether the relationship observed in ambulatory populations, surgical cohorts, and medical wards extends to the ICU environment remains unclear. The critically ill differ from these populations in several aspects, as they have a higher baseline comorbidity burden, exposure to multiple competing risk factors, and routine thromboprophylaxis in most cases. The substantial overlap between CVD, comorbidities, and obesity raises the possibility that previously reported associations may, in part, reflect shared confounding rather than independent venous wall pathology [23].

In this study, the Medical Information Mart for Intensive Care-IV (MIMIC-IV) database was chosen to examine the independent association between CVD and in-hospital VTE in adult ICU patients. Further investigation was performed to assess whether the relationship varied with the severity of venous disease, whether thromboprophylaxis differed between CVD and non-CVD patients, and whether the observed associations were robust to alternative analytical approaches.

## 2. Materials and Methods

A retrospective observational cohort study was conducted using the Medical Information Mart for Intensive Care-IV (MIMIC-IV) database, version 3.1 [24]. MIMIC-IV is a publicly available, deidentified critical care database derived from electronic health record data of adult patients admitted to intensive care units at the Beth Israel Deaconess Medical Center in Boston, Massachusetts, between 2008 and 2022. The authors completed the required PhysioNet credentialing process to access the data.

Eligible patients were adults (≥18 years) admitted to an ICU for the first time during their exposure to the database. Only each patient’s first ICU stay was considered to avoid within-patient clustering. An ICU length of stay of at least 48 h and survival through the first 48 h of ICU admission were required to ensure sufficient exposure time for the development of in-hospital VTE.

Patients with VTE present on admission (defined as a primary discharge diagnosis of DVT or PE), patients with a history of prior VTE or postthrombotic syndrome (identified by ICD-10 codes I87.0 or Z86.711/Z86.718, ICD-9 codes 459.1x or V12.51, or any DVT/PE code recorded in a prior MIMIC admission for the same patient), those receiving therapeutic-dose anticoagulation at baseline (defined as any prescription of warfarin, apixaban, rivaroxaban, dabigatran, edoxaban, or fondaparinux within the first 24 h of admission), and those with diagnoses of pregnancy, childbirth, or the puerperium (ICD-10 chapter O and selected Z codes; ICD-9 codes 630–679) were excluded.

The exposure of interest was chronic venous disease (CVD), defined in the CEAP framework as the full spectrum of chronic venous disorders of the lower limbs [9,10]. CVD was ascertained by the presence of any qualifying ICD code during the index admission or any prior MIMIC admission for the same patient. Qualifying codes were ICD-10 I83.x (varicose veins of lower extremities), I87.2 (chronic venous insufficiency, peripheral), I87.3x (chronic venous hypertension, idiopathic), and I87.9 (disorder of vein, unspecified); and ICD-9 454.x (varicose veins of lower extremities) and 459.81 (venous insufficiency, unspecified). These codes span CEAP classes C2 to C6. Except for the complication modifiers described below, they provide no information on edema or skin changes. I83.9 and 454.9 denote varicose veins without complications, that is, CEAP C2. The complete code list with per-code patient counts is provided in Supplementary Table S2. Because MIMIC-IV records no timestamp for diagnostic coding, exposure documented during the index admission cannot be positioned relative to the VTE event, and a diagnosis coded during that admission may in principle post-date the outcome; to bound this, a sensitivity analysis repeated the primary model under a stricter exposure definition restricted to codes recorded on the index admission and excluding the unspecified I87.9 and 459.81 codes.

The code set therefore identifies CVD rather than chronic venous insufficiency in the strict sense of C3–C6, and the exposure is reported as CVD. For a secondary analysis of disease severity, exposed patients were classified as having uncomplicated CVD (varicose veins, venous insufficiency, or chronic venous hypertension coded without ulceration or inflammation, approximating CEAP C2–C3) or complicated CVD (varicose veins or venous hypertension coded with ulceration, inflammation, or other complications, approximating CEAP C4b–C6). This dichotomy is the closest approximation to CEAP severity that administrative coding permits. It does not reproduce C-class assignment, and no direct clinical or duplex marker of venous disease severity was available in the data source.

The primary outcome was the presence of in-hospital VTE (DVT or PE) during the index hospitalization. PE was identified by ICD-10 I26.x or ICD-9 415.1x. DVT was identified by codes for acute thrombosis of deep veins: ICD-10 I82.0, I82.210, I82.220, I82.290, I82.3, I82.4x, I82.62x, I82.890, I82.A1x, I82.B1x, and I82.C1x; and ICD-9 453.0, 453.2, 453.3, 453.4x, 453.82, 453.84–453.87, 453.89, and 453.9. Codes for superficial venous thromboses (ICD-10 I82.61x, I82.81x; ICD-9 453.6, 453.81) and chronic venous thromboses (ICD-10 I82.5x, I82.7x; ICD-9 453.5x, 453.7x) were explicitly excluded. PE and DVT were also analyzed separately as secondary outcomes.

MIMIC-IV does not include a present-on-admission flag. To enrich the outcome for incident, hospital-acquired events, four restrictions were applied a priori: patients whose primary discharge diagnosis was DVT or PE were excluded; patients carrying any prior VTE or post-thrombotic syndrome code, in the index admission or in any earlier MIMIC admission, were excluded; patients receiving therapeutic-dose anticoagulation within the first 24 h were excluded; and only VTE codes recorded in a secondary diagnosis position (sequence number > 1) in patients who remained in the ICU for at least 48 h, were counted as outcomes.

Demographic variables included age at first ICU admission and sex. Severity of illness was characterized using the Sequential Organ Failure Assessment (SOFA) score within the first 24 h and the Simplified Acute Physiology Score II (SAPS-II). Comorbidities were quantified using the Charlson Comorbidity Index (CCI) [25]. Body mass index (BMI) was computed from first-day height and weight measurements when both were available.

Exposures during admission included invasive mechanical ventilation, vasopressor support, and renal replacement therapy. Thromboprophylaxis variables were extracted from the prescriptions table and chart documentation. Pharmacological prophylaxis was defined as any subcutaneously administered unfractionated heparin, low-molecular-weight heparin, or fondaparinux during the ICU stay, excluding prescriptions labeled as treatment-dose enoxaparin. Mechanical prophylaxis was identified from chart events.

### Statistical Analysis

Continuous variables were summarized as the mean with standard deviation when approximately normally distributed and as the median with the interquartile range otherwise; categorical variables were summarized as frequencies and percentages. Between-group comparisons were performed using *t*-tests, Mann–Whitney U tests, or chi-square tests as appropriate. Standardized mean differences (SMDs) were calculated to assess the magnitude of imbalance, with values > 0.10 considered clinically meaningful following the convention of Austin [26]. The functional form of age was examined with restricted cubic splines (four knots at the 5th, 35th, 65th, and 95th centiles), and departure from linearity was assessed by a likelihood-ratio test.

The primary analysis was a multivariable binary logistic regression for the composite VTE outcome, with CVD status as the exposure of interest and adjustment for age, sex, SAPS-II, Charlson Comorbidity Index, mechanical ventilation, and vasopressor support. The covariate set was specified a priori to represent three distinct domains: demography, acute physiological severity, and chronic comorbidity burden. Mechanical ventilation and vasopressor support were included as markers of admission acuity; as MIMIC-IV does not timestamp these exposures relative to the VTE event, they are treated as proxies for baseline severity rather than as strictly pre-outcome confounders. SAPS-II was chosen as the single index of acute severity in preference to SOFA because the respiratory and cardiovascular components of SOFA are defined in part by mechanical ventilation and vasopressor administration, which enter the model as explicit covariates. Separate models were fitted for PE and DVT.

BMI was not included in the primary model because of its substantial missingness (38.7%). BMI was instead added in a sensitivity analysis using multiple imputation by chained equations under a missing-at-random assumption, with five imputations and ten iterations per imputation [27]. The missing-at-random assumption cannot be verified against the underlying reason for non-documentation, and the BMI-adjusted estimate is therefore treated as supportive rather than definitive. A complete-case analysis restricted to patients with recorded BMI was additionally performed, and BMI availability was tabulated by CVD status and by VTE outcome (Supplementary Table S3). The imputation model included the exposure, the outcome, and all primary-model covariates. To address baseline imbalance, propensity score matching was performed as a sensitivity analysis. The propensity score for CVD status was estimated using logistic regression with the primary covariate set, and matching was performed 1:3 by nearest-neighbor with a caliper of 0.2 standard deviations of the logit propensity score. Balance after matching was assessed using post-match SMDs. Covariate balance before and after matching is displayed as a Love plot, and VTE events are reported separately for each matched group.

The severity analysis compared uncomplicated and complicated CVD with the unexposed group in a single model using the primary covariate set, with a trend test treating severity as an ordinal variable fitted separately. Because the complicated CVD stratum contained few outcome events relative to the number of model parameters, estimates for that stratum are subject to sparse-data bias, which drives maximum-likelihood estimates away from the null and renders their Wald confidence intervals unreliable [28]. The severity analysis was accordingly specified as exploratory, and its estimates are not used to support inference about a dose–response relationship.

Effect modification was evaluated with formal interaction tests using likelihood-ratio tests of nested logistic regression models. To assess robustness to unmeasured confounding, E-values for the primary point estimate and the limit of its 95% confidence interval closest to the null were computed after converting odds ratios to approximate risk ratios by the square-root transformation [29]. Cohort extraction was performed in PostgreSQL 17 (https://www.postgresql.org/docs/17/index.html accessed on 1 August 2026), using SQL. Statistical analyses were performed in R version 4.5 (R Foundation for Statistical Computing, Vienna, Austria). All *p*-values are two-sided, with significance set at *p* < 0.05.

## 3. Results

Of the initial 30,994 patients who met the inclusion criteria, 2253 were excluded, leaving a final analytic cohort of 28,741 patients. Of these, 544 (1.9%) had documented CVD, and 28,197 did not. CVD patients were on average 5.6 years older than their non-CVD counterparts (mean age 70.6 vs. 65.0 years; SMD 0.37), had a higher BMI (median BMI 33.0 vs. 28.0 kg/m^2^; SMD 0.66), and were substantially more comorbid (median Charlson Comorbidity Index 6.0 vs. 5.0; SMD 0.47). Prevalences of major comorbid conditions were higher in the CVD arm, including congestive heart failure (55.0% vs. 26.5%), chronic kidney disease (36.2% vs. 18.6%), chronic pulmonary disease (37.7% vs. 23.6%), and diabetes mellitus (40.8% vs. 28.7%) (*p* = 0.001 for all comparisons). The proportion of patients requiring mechanical ventilation was similar between groups (55.0% vs. 55.2%, *p* = 0.948), and vasopressor support was more frequent in the CVD arm (53.5% vs. 40.4%, *p* = 0.001) (Table 1).

CVD patients received less pharmacological prophylaxis than non-CVD patients (61.2% vs. 66.0%, *p* = 0.023) and less mechanical prophylaxis (5.5% vs. 9.7%, *p* = 0.001). Combined exposure to any pharmacological or mechanical prophylaxis was also lower in the CVD arm (63.1% vs. 68.7%, *p* = 0.005). Among CVD patients who received pharmacological prophylaxis, treatment was initiated earlier (median 6.5 h vs. 10.0 h from ICU admission, *p* = 0.001), and a slightly higher proportion received any prophylactic dose within the first 24 h than non-CVD patients (44.7% vs. 41.4%, *p* = 0.134). Composite categories showed that 36.9% of CVD patients received no prophylaxis, compared with 31.3% of non-CVD patients (Table 2).

The unadjusted incidence of any VTE was 7.0% among CVD patients vs. 6.2% among those without CVD (unadjusted OR 1.14, 95% CI 0.82–1.59, *p* = 0.437). After adjustment for age, sex, SAPS-II, Charlson Comorbidity Index, mechanical ventilation, and vasopressor support, the adjusted OR was 1.10 (95% CI 0.77–1.52, *p* = 0.571). When BMI was incorporated via multiple imputation, the adjusted association further attenuated, yielding an OR of 1.03 (95% CI 0.74–1.45, *p* = 0.849). Propensity score matching yielded a matched cohort of 2176 patients (544 CVD; 1632 non-CVD controls). Post-match SMDs were all below 0.05, indicating covariate balance. In the matched cohort, the OR for composite VTE was 1.24 (95% CI 0.84–1.84, *p* = 0.275). Outcome-specific analyses for PE and DVT produced similar results: the PE estimate (adjusted OR 1.31, 95% CI 0.83–2.07) was directionally elevated but not statistically significant, while the DVT estimate (adjusted OR 0.98, 95% CI 0.65–1.49) was essentially null (Table 3). Several prespecified sensitivity analyses supported the primary result. A complete-case analysis including BMI (*n* = 17,632) yielded an adjusted OR of 1.18 (95% CI 0.80–1.75, *p* = 0.404); BMI availability was 61.9% in CVD and 61.3% in non-CVD patients and was modestly higher among patients with VTE (68.0% vs. 60.9%; Supplementary Table S3). Restricting exposure to the stricter index-admission definition that excluded the unspecified I87.9 and 459.81 codes left the estimate unchanged (adjusted OR 1.05, 95% CI 0.70–1.58, *p* = 0.822). Modeling age with restricted cubic splines showed no departure from linearity (likelihood-ratio *p* = 0.24), and the CVD estimate was unchanged (adjusted OR 1.10). In the propensity-matched cohort all standardized mean differences fell below 0.05 (Supplementary Figure S1), and VTE occurred in 7.0% of matched CVD patients versus 5.8% of matched controls.

After adjustment in the multiple regression model, CVD was not significantly associated with VTE (aOR 1.10, *p* = 0.571). The strongest independent predictor was mechanical ventilation (aOR 1.38, 95% CI 1.23–1.55, *p* = 0.001), followed by Charlson Comorbidity Index (aOR 1.07 per point, *p* = 0.001), SAPS-II (aOR 1.01 per point, *p* = 0.001), and female sex (aOR 1.12, *p* = 0.022). Age was inversely associated with VTE risk (aOR 0.98 per year, *p* = 0.001). Vasopressor support showed a non-significant trend (aOR 1.11, *p* = 0.059) (Table 4).

Predictors of receiving any pharmacological prophylaxis were evaluated. CVD status showed a non-significant association with reduced prophylaxis (aOR 0.88, 95% CI 0.74–1.06, *p* = 0.172). Independent of severity and comorbidity, female sex was associated with higher odds of prophylaxis (aOR 1.29, *p* = 0.001), and vasopressor support was associated with markedly lower odds (aOR 0.75, *p* = 0.001). SAPS-II showed a statistically significant but numerically negligible association with prophylaxis receipt (aOR 1.00 per point, *p* = 0.019), and the Charlson Comorbidity Index was not associated (aOR 1.00 per point, *p* = 0.407) (Table 5).

Crude VTE rates across CVD severity categories were 6.2% in patients without CVD, 6.4% in those with uncomplicated CVD (adjusted OR vs. no CVD 1.01, 95% CI 0.69–1.42), and 16.7% in patients with complicated CVD (adjusted OR 2.90, 95% CI 1.09–7.08, *p* = 0.032). The complicated CVD stratum comprised 30 patients with 5 events, fewer than one outcome event per model parameter, so this estimate and its confidence interval are considered unstable. The trend test treating severity as an ordinal variable was not significant (aOR 1.17 per severity level, *p* = 0.31), so the ordering of the three point estimates does not constitute evidence of a dose–response relationship. These severity estimates are reported as exploratory. Formal interaction tests for effect modification by age (≥65 vs. <65), sex, and active malignancy were non-significant (all *p* > 0.24), while the CVD × mechanical ventilation interaction approached statistical significance (*p* = 0.062).

A cancer-stratified descriptive analysis did not show any statistically significant observations. E-value sensitivity analyses confirmed that the 95% confidence intervals for all primary associations already included the null and that the point E-values were modest (1.14–1.55), indicating that any residual association after adjustment could be explained by a weak unmeasured confounder.

## 4. Discussion

In this retrospective cohort study of 28,741 critically ill adults derived from the MIMIC-IV database, chronic venous disease was not independently associated with in-hospital venous thromboembolism after adjustment for age, sex, severity scores, comorbidity burden, and ICU-specific treatment exposures. The crude association was modest and not statistically significant (OR 1.14, 95% CI 0.82–1.59), and it attenuated further when body mass index was incorporated via multiple imputation (OR 1.03, 95% CI 0.74–1.45). Because no association was present to begin with, this pattern is consistent with, but does not establish, confounding by obesity. In an exploratory severity analysis, patients with complicated CVD had a higher crude VTE rate than those without CVD, but that stratum contained only 5 events, and the test for trend across severity categories was not significant.

Our findings stand in contrast to the largely positive published literature on CVD and VTE. The population-based study by Chang and colleagues reported hazard ratios of 5.30 for DVT and 1.73 for PE in patients with varicose veins compared with propensity-matched controls in the general Taiwanese population [18]. Other studies in orthopedic surgery and in hospitalized patients with COPD exacerbation have reported positive associations, although effect magnitudes have varied [19,21]. Two possible explanations may be stated for this difference. First, the critically ill population differs fundamentally from the other studied populations. In ambulatory and surgical cohorts, baseline VTE risk is comparatively low, and chronic venous wall pathology could manifest as an incremental risk factor. In the ICU, exposure to many potent thrombogenic factors is present for the majority of patients, including immobility, central venous catheters, mechanical ventilation, coagulopathy, vasopressor-induced microcirculatory changes, and invasive interventions [3,30]. Against this background, a chronic venous condition may add relatively little independent risk.

Secondly, CVD patients in our cohort had a higher BMI than their counterparts. When BMI was incorporated through multiple imputation, the adjusted OR for CVD attenuated from 1.10 to 1.03, while BMI itself emerged as an independent VTE predictor. Obesity is a well-established VTE risk factor through multiple mechanisms, including increased intra-abdominal pressure, venous stasis, adipokine-mediated inflammation, and hypercoagulability [23,31]. The overlap between obesity and CVD in the general population raises the possibility that previously reported associations between varicose veins and VTE partly reflect shared confounding by obesity rather than an independent thrombogenic contribution of venous wall pathology [14]. The crude CVD–VTE association in our cohort was not statistically significant, so the attenuation from 1.10 to 1.03 is a difference between two null estimates rather than the dissolution of a real association; because BMI was missing in more than a third of the cohort, the BMI-adjusted estimate rests on a missing-at-random assumption that cannot be verified. What these data support is the narrower claim that BMI is an independent predictor of VTE in this population and that CVD carries no detectable independent risk once BMI is accounted for. Whether obesity explains the associations reported in other populations is a question our data cannot settle.

Although the overall CVD–VTE association was null, the ordering of the severity estimates warrants comment, with the caveat that this is exploratory and should not be interpreted as a dose–response relationship. Patients with complicated CVD, whose codes indicate ulceration or inflammation and therefore approximate CEAP C4b–C6, had a crude VTE rate of 16.7%, compared with 6.2% in patients without CVD and 6.4% in those with uncomplicated CVD. The adjusted odds ratio of 2.90 (95% CI 1.09–7.08) is based on 5 events in 30 patients within a seven-parameter model, a configuration in which maximum-likelihood estimates are known to be biased away from the null and their Wald intervals unreliable [28]. Its nominal significance should therefore not be taken as evidence of an effect, and the formal test for trend across severity categories was not significant (*p* = 0.31). The severity analysis therefore generates a hypothesis rather than supporting a conclusion. That hypothesis is biologically reasonable, since ulcerated and inflamed venous walls would be expected to carry greater thrombogenic potential than asymptomatic varicosities [15,32], and the relationship between venous disease severity and thrombotic risk has been reviewed in detail elsewhere [22]. It is equally compatible with residual confounding because complicated venous disease marks immobility, chronic inflammation, and general debility, none of which our covariates capture. The CVD × mechanical ventilation interaction likewise approached but did not reach significance (*p* = 0.062).

CVD patients received less thromboprophylaxis than non-CVD patients, despite a higher overall risk profile, another observation that warrants further investigation. Although the association did not reach statistical significance in the fully adjusted model (aOR 0.88, *p* = 0.172), the consistent directional pattern across pharmacological, mechanical, and any-prophylaxis categories suggests that CVD status does not prompt aggressive preventive interventions in routine ICU practice. This is somewhat surprising given that varicose veins carry an explicit point in the Caprini risk assessment model [7]. They are not, however, among the eleven items of the Padua Prediction Score [8], which is the instrument more commonly applied to medical inpatients, nor among the seven variables of the IMPROVE (International Medical Prevention Registry on Venous Thromboembolism) VTE risk score, the most widely used VTE risk assessment model in hospitalized medical patients globally, which is of direct relevance to the present cohort because admission to an intensive care unit is itself one of those seven variables [33]. A risk factor that does not appear in these scores in routine use will not enter the assessment. The reasons cannot be determined from these data, and the following are hypotheses only: chronic venous disease may be regarded as a stable condition unrelated to acute thrombotic risk; competing prescribing constraints, such as bleeding risk, recent invasive procedures, and hemodynamic instability, may dominate the decision; or CVD may simply be documented inconsistently at ICU admission and therefore never be considered. The observation that patients receiving vasopressors were less likely to receive pharmacological prophylaxis (aOR 0.75, *p* = 0.001) is consistent with the second of these, in that acute hemodynamic concerns appear to displace attention from chronic risk factors. Patients with CVD were substantially more likely to receive vasopressors (53.5% vs. 40.4%, *p* = 0.001), and vasopressor exposure independently predicted lower odds of prophylaxis. The attenuation of the crude prophylaxis difference after adjustment is therefore consistent with a shortfall driven partly by co-occurring hemodynamic instability rather than by CVD status itself.

Several limitations should be acknowledged. First, the exposure assessment relied on ICD coding, which is well known to under-detect chronic conditions, particularly during admissions dominated by acute diagnoses [25]. Even with cross-admission look-back, many ICU patients with subclinical or under-documented venous disease were likely misclassified as unexposed; the observed exposure prevalence of 1.9% lies an order of magnitude below the population prevalence of chronic venous disease and is itself evidence of substantial under-ascertainment. Administrative codes also cannot assign a CEAP class. They record whether varicose veins or venous insufficiency were documented and whether ulceration or inflammation was noted, but they carry no information on edema or skin changes, so the severity contrast reported here approximates C2–C3 versus C4b–C6 rather than reproducing a C-class analysis. Exposure misclassification of this kind, if non-differential, would bias estimates toward the null and could mask a true association. Second, MIMIC-IV does not contain a present-on-admission indicator, so some secondary VTE codes may reflect events present at admission rather than hospital-acquired ones. Four design features mitigate this: exclusion of patients whose primary discharge diagnosis was DVT or PE; exclusion of any patient carrying a prior VTE or post-thrombotic syndrome code in the index or any earlier admission; exclusion of patients on therapeutic anticoagulation within the first 24 h; and the requirement for at least 48 h of ICU stay. Third, the data derive from a single tertiary academic center, which limits external generalizability, particularly for the prophylaxis findings, since the prescribing approach is often institution-specific. Fourth, the complicated CVD stratum contained 30 patients and 5 events. With fewer than one event per model parameter, the estimate for that stratum is subject to sparse-data bias and its Wald confidence interval is unreliable [28]; no inference about a severity gradient should be drawn from it. Adequately powered estimation for advanced venous disease will require pooling across cohorts rather than reanalysis of these data. Fifth, SAPS-II incorporates age within its own calculation, so the age coefficient in the primary model represents the residual association of age conditional on physiological severity rather than the total effect of age on VTE risk; the inverse point estimate observed for age should be read in that light and may additionally reflect the competing risk of early death and less intensive diagnostic imaging in the oldest patients. Sixth, BMI was missing in 38.7% of the cohort, and multiple imputation assumes the data are missing at random conditional on the observed variables. That assumption cannot be tested here, and the BMI-adjusted estimates are accordingly supportive rather than definitive. Another, more fundamental issue is that the analysis rests on administrative codes that carry no event timing. The sequence of discharge diagnoses does not establish when in-hospital VTE occurred, so events cannot be confirmed to have arisen after the 48 h landmark used for eligibility, and the binary outcome model does not account for differential time at risk. For the same reason, venous disease coded during the index admission cannot be ordered relative to the VTE event and may in a minority of cases represent reverse temporal classification, although the stricter index-admission definition gave concordant results. Mechanical ventilation and vasopressor support are likewise not timestamped relative to the outcome and are therefore adjusted as severity proxies rather than as confirmed pre-outcome confounders. Finally, the thromboprophylaxis analysis shares these timing limitations and is additionally uninformed by data on bleeding risk and contraindications; it is descriptive and should not be read as evidence of a gap in care.

These limitations also define a specific agenda for future work. First, exposure ascertainment must move beyond administrative coding: linkage to clinical documentation, duplex ultrasonography, or prospective CEAP classification at ICU admission would enable C-class-specific analysis that ICD codes cannot support. Second, because advanced venous disease is uncommon in unselected ICU populations, adequately powered estimates for C4b–C6 patients will require multicenter pooling across critical care databases. Third, the reasons patients with chronic venous disease received less thromboprophylaxis cannot be recovered from structured data. Qualitative or mixed-methods work examining clinician reasoning and documentation practices would be more informative on that question than any further database analysis. Finally, external validation across health systems with different prophylaxis cultures is needed before the prophylaxis findings, in particular, can be generalized beyond a single tertiary academic center.

## 5. Conclusions

In an unselected cohort of 28,741 critically ill adults, chronic venous disease was not independently associated with in-hospital venous thromboembolism. The crude association was modest and not statistically significant, and it attenuated further once body mass index was accounted for, a pattern consistent with the hypothesis that previously reported associations between varicose veins and VTE in ambulatory and surgical populations partly reflect shared confounding by obesity in a setting where multiple competing acute thrombogenic stimuli predominate. An exploratory analysis of disease severity suggested a possible elevated risk in complicated CVD, but with 5 events in 30 patients and a non-significant test for trend, this remains a hypothesis to be tested in cohorts with prospective CEAP classification rather than a finding. CVD patients received less thromboprophylaxis than their counterparts without CVD despite a higher baseline risk burden, although this difference did not persist after adjustment and may partly reflect their greater exposure to vasopressors, which independently predicted lower odds of prophylaxis.

## Figures and Tables

**Table 1:** Baseline characteristics of the cohort.

Demographic Characteristic		No CVD (*n* = 28,197)	CVD (*n* = 544)	SMD	*p*-Value
Age, years, mean (SD)		65.0 (16.6)	70.6 (13.4)	0.37	0.001
Gender (*n*, %)	Female	12,163 (43.1)	231 (42.5)	0.01	0.787
	Male	16,034 (56.9)	313 (57.5)		
BMI (kg/m^2^, median, IQR)		28.0 (24.2–32.8)	33.0 (27.7–39.9)	0.66	0.001
*ICU course*					
SOFA score (median, IQR)		4.0 (2.0–7.0)	6.0 (3.0–8.0)	0.28	0.001
SAPS-II (median, IQR)		36.0 (28.0–45.0)	40.0 (32.0–50.0)	0.3	0.001
Admission length (days, median, IQR)		3.91 (2.73–6.88)	3.96 (2.78–6.10)	0.09	0.399
Mechanical ventilation, *n* (%)		15,564 (55.2)	299 (55.0)	0.01	0.948
Vasopressor support, *n* (%)		11,390 (40.4)	291 (53.5)	0.27	0.001
Renal replacement therapy, *n* (%)		1587 (5.6)	43 (7.9)	0.09	0.029
*Comorbidities (n, %)*					
Congestive heart failure		7483 (26.5)	299 (55.0)	0.6	0.001
Chronic pulmonary disease		6643 (23.6)	205 (37.7)	0.31	0.001
Diabetes mellitus		8104 (28.7)	222 (40.8)	0.26	0.001
Chronic kidney disease		5237 (18.6)	197 (36.2)	0.4	0.001
Peripheral vascular disease		3351 (11.9)	104 (19.1)	0.2	0.001
Active malignancy		4011 (14.2)	67 (12.3)	0.06	0.229
CCI (median, IQR)		5.0 (3.0–7.0)	6.0 (5.0–8.0)	0.47	0.001
*In-hospital outcomes (n, %)*					
Venous Thromboembolism		1741 (6.2)	38 (7.0)	0.03	0.492
Pulmonary embolism		817 (2.9)	20 (3.7)	0.04	0.346
Deep vein thrombosis		1165 (4.1)	24 (4.4)	0.01	0.829
In-hospital mortality		3725 (13.2)	72 (13.2)	0.01	0.740

SMD: standardized mean difference; CVD: chronic venous disease; SOFA: Sequential Organ Failure Assessment; SAPS-II: Simplified Acute Physiology Score II; BMI: body mass index; ICU: intensive care unit; CCI: Charlson Comorbidity Index. *p* values were obtained from *t*-tests, Mann–Whitney U tests, or chi-square tests as appropriate.

**Table 2:** Thromboprophylaxis patterns during the intensive care admission.

Prophylaxis Methods (*n*, %)	No CVD (*n* = 28,197)	CVD (*n* = 544)	SMD	*p*-Value
Any pharmacological prophylaxis	18,598 (66.0)	333 (61.2)	0.1	0.023
Early (≤24 h) prophylaxis	11,669 (41.4)	243 (44.7)	0.07	0.134
Hours to first dose (median, IQR)	10.0 (2.5–42.7)	6.5 (2.3–25.2)	0.2	0.001
Days of prophylaxis (median, IQR)	1.0 (0.0–1.0)	1.0 (0.0–1.0)	0.1	0.028
Mechanical prophylaxis	2735 (9.7)	30 (5.5)	0.16	0.001
*Composite prophylaxis category, n (%)*				
None	8819 (31.3)	201 (36.9)	N/A	
Mechanical only	780 (2.8)	10 (1.8)		
Pharmacological only	16,643 (59.0)	313 (57.5)		
Combined pharmacological + mechanical	1955 (6.9)	20 (3.7)	0.18	0.001
Any prophylaxis (pharmacological or mechanical)	19,378 (68.7)	343 (63.1)	0.12	0.005

Pharmacological prophylaxis was defined as any subcutaneously administered heparin, low-molecular-weight heparin, or fondaparinux during the ICU stay, excluding prescriptions labeled as treatment-dose enoxaparin. Mechanical prophylaxis was ascertained from structured chart documentation of sequential or intermittent pneumatic compression devices. SMD: standardized mean difference; CVD: chronic venous disease “N/A: not applicable” should be added.

**Table 3:** Odds ratios for in-hospital venous thromboembolism.

Outcome and Analysis	Odds Ratio (95% CI)	*n*/Events	*p*-Value
*VTE composite (DVT or PE)*			
Unadjusted	1.14 (0.82–1.59)	28,741/1779	0.437
Adjusted ^1^	1.10 (0.77–1.52)	28,741/1779	0.571
Adjusted with BMI ^2^	1.03 (0.74–1.45)	28,741/1779	0.849
Propensity score matched (1:3) ^3^	1.24 (0.84–1.84)	2176/—	0.275
*Pulmonary embolism*			
Unadjusted	1.28 (0.81–2.01)	28,741/837	0.286
Adjusted ^1^	1.31 (0.83–2.07)	28,741/837	0.239
Propensity score matched (1:3) ^3^	1.41 (0.82–2.42)	2176/—	0.212
*Deep vein thrombosis*			
Unadjusted	1.07 (0.71–1.62)	28,741/1189	0.745
Adjusted ^1^	0.98 (0.65–1.49)	28,741/1189	0.937
Propensity score matched (1:3) ^3^	1.21 (0.75–1.96)	2176/—	0.441
*Subgroup analyses (VTE composite, adjusted ^1^* *)*			
Age < 65 years	1.14 (0.65–1.99)	12,738/162	0.648
Age ≥ 65 years	1.09 (0.71–1.66)	16,003/382	0.696
Male sex	0.96 (0.60–1.53)	16,347/313	0.852
Female sex	1.32 (0.82–2.14)	12,394/231	0.252
No active malignancy	1.18 (0.81–1.72)	24,663/477	0.390
Mechanically ventilated	0.87 (0.55–1.38)	15,863/299	0.56

^1^ Primary multivariable logistic regression model adjusted for age, sex, SAPS-II, Charlson Comorbidity Index, mechanical ventilation, and vasopressor support. ^2^ Sensitivity analysis using multiple imputation (m = 5) for missing BMI values. ^3^ Propensity score matching (1:3 nearest-neighbor, caliper 0.2 SD of logit propensity score) on the primary covariate set; all post-match standardized mean differences <0.05. CI: confidence interval; BMI: body mass index.

**Table 4:** Multivariable logistic regression model for in-hospital venous thromboembolism.

Variable	Adjusted Odds Ratio (95% CI)	*p*-Value
Chronic venous disease	1.10 (0.77–1.52)	0.571
Age	0.98 (0.97–0.98)	0.001
Female sex	1.12 (1.02–1.24)	0.022
SAPS-II	1.01 (1.01–1.02)	0.001
Charlson Comorbidity Index	1.07 (1.05–1.09)	0.001
Mechanical ventilation	1.38 (1.23–1.55)	0.001
Vasopressor support	1.11 (1.00–1.24)	0.059

The model included 28,741 patients with 1779 events. Estimates are odds ratios with 95% confidence intervals from binary logistic regression. CI: confidence interval; SAPS-II, Simplified Acute Physiology Score II.

**Table 5:** Predictors of pharmacological thromboprophylaxis during admission.

Variable	Adjusted Odds Ratio (95% CI)	*p*-Value
Chronic venous disease	0.88 (0.74–1.06)	0.172
Age	0.99 (0.99–0.99)	0.001
Female sex	1.29 (1.23–1.36)	0.001
SAPS-II	1.00 (1.00–1.00)	0.019
Charlson Comorbidity Index	1.00 (0.99–1.02)	0.407
Mechanical ventilation	1.05 (0.99–1.11)	0.119
Vasopressor support	0.75 (0.71–0.79)	0.001

CI: confidence interval; SAPS-II: Simplified Acute Physiology Score II.

## Data Availability

The data analyzed in this study are publicly available through the Medical Information Mart for Intensive Care-IV (MIMIC-IV) database (https://physionet.org/content/mimiciv/ accessed on 28 May 2026) under credentialed access. Analytical code is available from the corresponding author upon reasonable request.

## Supplementary Materials

The following supporting information can be downloaded at https://www.mdpi.com/article/10.3390/jcm15176695/s1. Table S1: Disease severity, interaction, sensitivity, and stratified analyses of venous thromboembolism; Table S2: Qualifying ICD diagnosis codes for chronic venous disease; Table S3: Availability of body mass index by chronic venous disease status and by venous thromboembolism outcome; Figure S1: Covariate balance before and after 1:3 propensity-score matching (Love plot).

## Abbreviations

The following abbreviations are used in this manuscript:

AECOPD	Acute exacerbation of chronic obstructive pulmonary disease
aOR	Adjusted odds ratio
BMI	Body mass index
CCI	Charlson Comorbidity Index
CEAP	Clinical-Etiological-Anatomical-Pathophysiological
CI	Confidence interval
COPD	Chronic obstructive pulmonary disease
CVD	Chronic venous disease
CVI	Chronic venous insufficiency
DVT	Deep vein thrombosis
HIPAA	Health Insurance Portability and Accountability Act
ICD	International Classification of Diseases
ICU	Intensive care unit
IMPROVE	International Medical Prevention Registry on Venous Thromboembolism
MIMIC-IV	Medical Information Mart for Intensive Care-IV
OR	Odds ratio
PE	Pulmonary embolism
SAPS-II	Simplified Acute Physiology Score II
SMD	Standardized mean difference
SOFA	Sequential Organ Failure Assessment
SQL	Structured Query Language
VTE	Venous thromboembolism
