# Supplementary material for: Chronic Venous Disease and In-Hospital Venous Thromboembolism in Critically Ill Patients: A Retrospective Cohort Study of the MIMIC-IV Database
Source: J Clin Med. 2026 Aug 28;15(17):6695. doi: 10.3390/jcm15176695 (PMC13565820; doi:10.3390/jcm15176695)

Supplementary Materials

Supplementary Table S1: Disease severity, interaction, sensitivity, and stratified analyses of venous thromboembolism.

Analysis	Estimate (95% CI)	p	n / events
**CVD severity strata — VTE composite (aOR[1])**			
No CVD (reference)	1.00 (reference)	—	28,197 / 1,741 (6.2%)
Uncomplicated CVD	1.01 (0.69–1.42)	0.968	514 / 33 (6.4%)
Complicated CVD	2.90 (1.09–7.08)	0.032	30 / 5 (16.7%)
**Interaction tests[2] (likelihood-ratio χ^2, df = 1)**			
CVD × Age	χ^2 = 0.711	0.399	—
CVD × Sex	χ^2 = 1.338	0.247	—
CVD × Mechanical ventilation	χ^2 = 3.472	0.062	—
CVD × Active malignancy	χ^2 = 0.017	0.896	—
**Cancer-stratified CVD effect on VTE (aOR[1])**			
No active malignancy	1.18 (0.81–1.72)	0.390	24,663 / 1,371
Active malignancy	1.21 (0.57–2.56)	0.628	4,078 / 408
**E-value sensitivity analysis[3]**	**Estimate (95% CI)**	**E-value (point)**	**E-value (CI limit)**
Adjusted VTE composite (aOR[1])	1.10 (0.77–1.52)	1.28	1.00
Adjusted VTE composite, with BMI (aOR)	1.03 (0.74–1.45)	1.14	1.00
Adjusted PE (aOR[1])	1.31 (0.83–2.07)	1.55	1.00
PSM-matched VTE composite (OR)	1.24 (0.84–1.84)	1.47	1.00

[1]Adjusted for age, sex, SAPS-II, Charlson Comorbidity Index, mechanical ventilation, and vasopressor support. [2] Likelihood-ratio test comparing nested logistic regression models, with the interacting variable's main effect retained. [3] E-values quantify the minimum strength of association, on the risk-ratio scale, that an unmeasured confounder would need to have with both the exposure and the outcome to explain away the observed association. The point E-value refers to the point estimate and the CI-limit E-value to the confidence limit closest to the null; a value of 1.00 indicates that the interval already includes the null. Odds ratios were converted to approximate risk ratios by the square-root transformation before E-values were calculated. **CVD:** Chronic Venous Disease; **VTE:** Venous Thromboembolism; **OR:** odds ratio; **aOR:** adjusted odds ratio; **CI:** confidence interval; **BMI:** Body Mass Index; **PE:** Pulmonary embolism; **PSM:** propensity score matching.

Supplementary Table S2: Qualifying ICD diagnosis codes for chronic venous disease

ICD version	Code	Description	Patients, n
ICD-9	459.81	Venous (peripheral) insufficiency, unspecified	367
ICD-10	I87.2	Venous insufficiency (chronic) (peripheral)	109
ICD-9	454.9	Asymptomatic varicose veins	35
ICD-10	I83.90	Asymptomatic varicose veins of unspecified lower extremity	15
ICD-10	I83.93	Asymptomatic varicose veins of bilateral lower extremities	9
ICD-10	I83.028	Varicose veins, left lower extremity, ulcer other part of lower leg	3
ICD-10	I83.029	Varicose veins, left lower extremity, ulcer of unspecified site	3
ICD-10	I83.228	Varicose veins, left lower extremity, ulcer other part and inflammation	3
ICD-9	454.0	Varicose veins of lower extremities with ulcer	2
ICD-10	I83.009	Varicose veins, unspecified lower extremity, ulcer of unspecified site	2
ICD-10	I83.018	Varicose veins, right lower extremity, ulcer other part of lower leg	2
ICD-10	I83.10	Varicose veins, unspecified lower extremity, with inflammation	2
ICD-10	I83.11	Varicose veins, right lower extremity, with inflammation	2
ICD-10	I83.12	Varicose veins, left lower extremity, with inflammation	2
ICD-10	I83.218	Varicose veins, right lower extremity, ulcer other part and inflammation	2
ICD-10	I83.223	Varicose veins, left lower extremity, ulcer of ankle and inflammation	2
ICD-10	I83.813	Varicose veins, bilateral lower extremities, with pain	2
ICD-9	454.2	Varicose veins of lower extremities with ulcer and inflammation	1
ICD-9	454.1	Varicose veins of lower extremities with inflammation	1
ICD-10	I83.019	Varicose veins, right lower extremity, ulcer of unspecified site	1

Codes shown are those identified on the index admission of exposed patients. The full qualifying definition searched across the index and all prior MIMIC-IV admissions comprised ICD-10 I83.x, I87.2, I87.3x, and I87.9, and ICD-9 454.x and 459.81, spanning CEAP classes C2–C6. **ICD:** International Classification of Diseases.

Supplementary Table S3: Availability of body mass index by chronic venous disease status and by venous thromboembolism outcome.

Stratum	With recorded BMI, n	Total, n	Available, %
**By CVD status**			
CVD	337	544	61.9
No CVD	17,295	28,197	61.3
**By VTE outcome**			
VTE	1,210	1,779	68.0
No VTE	16,422	26,962	60.9
**By CVD status and VTE outcome**			
CVD, VTE	29	38	76.3
CVD, no VTE	308	506	60.9
No CVD, VTE	1,181	1,741	67.8
No CVD, no VTE	16,114	26,456	60.9

BMI was recorded for 17,632 of 28,741 patients (61.3%). Availability was similar across CVD status but modestly higher among patients with VTE. **BMI:** Body Mass Index; **CVD:** Chronic Venous Disease; **VTE:** Venous Thromboembolism.

Supplementary Figure S1. Covariate balance before and after 1:3 propensity-score matching (Love plot)

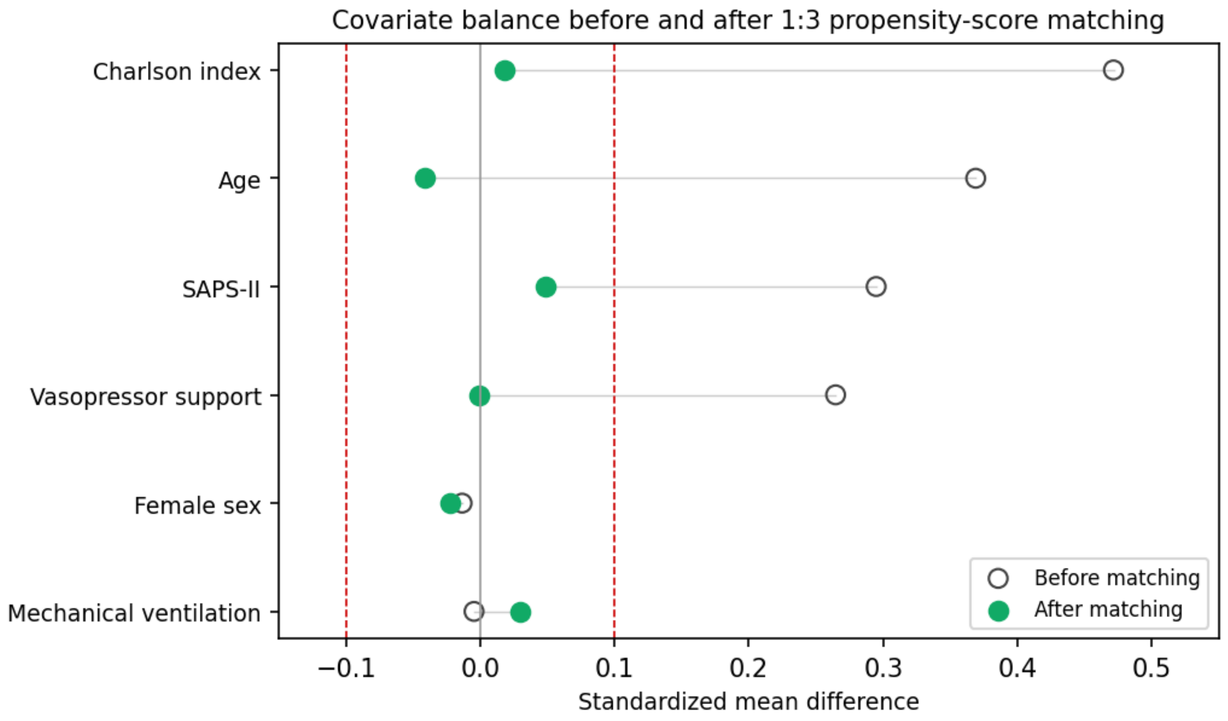

Supplement: Supplementary file 1 [file jcm-15-06695-s001.zip › jcm-4431082-supplementary.pdf]
